# *w*‐Type ions formed by electron transfer dissociation of Cys‐containing peptides investigated by infrared ion spectroscopy

**DOI:** 10.1002/jms.4298

**Published:** 2018-11-12

**Authors:** Lisanne J. M. Kempkes, Jonathan Martens, Giel Berden, Jos Oomens

**Affiliations:** ^1^ Radboud University, Institute for Molecules and Materials, FELIX Laboratory Nijmegen The Netherlands; ^2^ Van't Hoff Institute for Molecular Sciences University of Amsterdam Amsterdam The Netherlands

**Keywords:** cysteine, electron transfer dissociation, IRMPD spectroscopy, peptide dissociation, *w*‐type ions

## Abstract

In mass spectrometry‐based peptide sequencing, electron transfer dissociation (ETD) and electron capture dissociation (ECD) have become well‐established fragmentation methods complementary to collision‐induced dissociation. The dominant fragmentation pathways during ETD and ECD primarily involve the formation of *c*‐ and *z* ^•^‐type ions by cleavage of the peptide backbone at the N─C_α_ bond, although neutral losses from amino acid side chains have also been observed. Residue‐specific neutral side chain losses provide useful information when conducting database searching and de novo sequencing. Here, we use a combination of infrared ion spectroscopy and quantum‐chemical calculations to assign the structures of two ETD‐generated *w*‐type fragment ions. These ions are spontaneously formed from ETD‐generated *z* ^*•*^‐type fragments by neutral loss of 33 Da in peptides containing a cysteine residue. Analysis of the infrared ion spectra confirms that these *z* ^•^‐ions expel a thiol radical (SH^•^) and that a vinyl C═C group is formed at the cleavage site. *z* ^*•*^‐type fragments containing a Cys residue but not at the cleavage site do not spontaneously expel a thiol radical, but only upon additional collisional activation after ETD.

## 1 INTRODUCTION

**Scheme 1:**
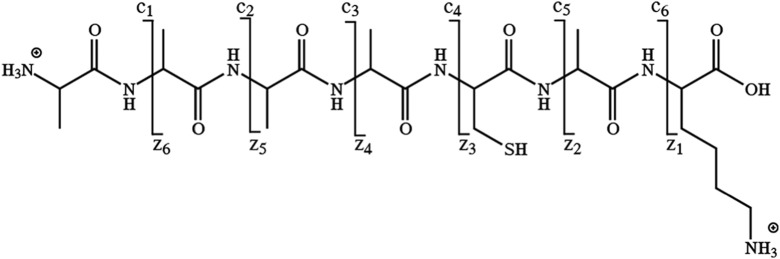
Overview of expected electron transfer dissociation sites cleaving the peptide backbone at one of the N─C_α_ bonds, generating N‐terminal *c*‐ions and/or C‐terminal *z*‐ions. The *z*‐ions are radical species

Mass spectrometry data in combination with bioinformatics algorithms form a key technology for peptide and protein sequencing, including the identification of posttranslational protein modifications.^1^, ^2^ Particularly with the advent of top‐down proteomics and the sequencing of posttranslational protein modifications, electron induced dissociation methods—either electron capture or electron transfer dissociation (ECD or ETD)—have become popular.^3^, ^4^ The dominant ETD and ECD fragmentation pathways involve cleavage of the N‐C_α_ bonds resulting in the formation of *c‐* and *z* ^*•*^‐type ions^5^, ^6^, ^7^, ^8^ (see Scheme 1). Neutral loss fragments including those from the residue side chains are also commonly observed.^9^, ^10^, ^11^, ^12^, ^13^, ^14^, ^15^, ^16^ Side chain losses can confirm sequence assignments, improve database matching scores, and can be useful in de novo sequencing^14^. It is therefore useful to train sequencing algorithms to recognize neutral loss fragments in addition to sequence ions.^17^ Neutral losses from residue side chains in radical ExD sequence ions have for instance been used to distinguish between leucine and isoleucine residues and to identify isomeric amino acid combinations.^17^, ^18^, ^19^, ^20^ On the other hand, extensive formation of nonsequence ions due to entire or partial side‐chain losses, especially when remote from the backbone cleavage site, may severely complicate database searching.^21^

Mechanistically, neutral loss from radical *z* ^*•*^‐type ions has been explained by several charge‐remote fragmentation pathways.^10^, ^21^, ^22^, ^23^ Migration of the radical may occur from the original site of cleavage at the C_α_ atom to the C_β_ and C_γ_ positions of the same residue or to C_α_, C_β_, and C_γ_ atoms in neighboring residues.^14^, ^18^, ^20^, ^21^, ^24^, ^25^ Subsequent expulsion of a neutral radical generates even‐electron fragment ions in the mass spectrum, which have been suggested to adopt a *w*‐type fragment ion structure by cleavage of the C_β_─C_γ_ bond of the N‐terminal residue and the formation of a double bond between the C_α_ and C_β_ atoms (direct formation). Alternatively, cyclic *u*‐type fragments may form as a result of bond formation between the N‐terminal C_α_ atom and the C_β_ atom of the adjacent residue.^16^, ^18^, ^21^, ^26^ A possible mechanism for the loss of the entire side chain involves H‐atom abstraction from the C_γ_ and subsequent α‐cleavage and expulsion of the side chain as an even‐electron neutral species, leaving the radical on the peptide backbone.^21^

Fung and Chan systematically investigated neutral loss product ions from ECD‐generated *z* ^*•*^‐type ions using the doubly charged peptides [RGGGXGGGR+2H]^2+^, where X denotes one of the 20 naturally occurring amino acid residues.^27^ Residues that were shown to undergo secondary loss of a neutral radical, thus leaving behind an even‐electron charged fragment, include among several others cysteine (Cys), the subject of investigation here. Even‐electron neutral losses from residue side chains were also reported for various other amino acid residues.^8^, ^24^, ^27^, ^28^

Cys‐specific ExD pathways have been discussed in several studies.^23^, ^27^, ^29^ For *z* ^*•*^‐type fragments from Cys‐containing peptides, secondary fragmentation by loss of a 33‐Da neutral fragment is common and is attributed to loss of a thiol radical (SH^•^). For z_6_‐ and larger *z* ^*•*^‐type ions, neutral loss of CH_2_S (46 Da) has also been observed.^27^ ETD on [AACAR+2H]^2+^ generated the z_4_‐ion (*m*/*z* 404) and the z_3_‐ion (*m*/*z* 333), where the intensity of the z_3_‐ion was significantly decreased by spontaneous loss of the SH radical, forming a secondary product at *m*/*z* 300.^29^ ECD studies on peptides containing modified cysteine residues, such as carboxymethylated or carbamidomethylated Cys residues, report neutral losses corresponding to the modified Cys side chain.^30^ The odd‐electron neutral loss of C_2_H_4_NOS^●^ (90 Da) has also been observed in a negative electron‐transfer dissociation process on a doubly negatively charged ion containing carbamidomethylated Cys residues.^31^

In related studies not actually involving ExD, the CID fragmentation behavior, and possible radical migration in open‐shell cations of Cys and Cys‐containing peptides have been studied by theoretical^31^, ^32^, ^33^ as well as experimental methods.^16^, ^23^, ^35^, ^36^, ^37^, ^38^, ^39^, ^40^ In experimental studies, CID‐induced loss of nitric oxide from protonated S‐nitrosocysteine is an efficient way to form radical cations of Cys and Cys‐containing peptides.^35^, ^36^ Hao and Gross^36^ showed that CID fragmentation of Cys‐containing peptide radicals yields fragmentation similar to ExD experiments. Fragmentation reactions of radical cations of Cys‐containing di‐ and tripeptides were found to be radical‐driven or charge directed, leading to losses of SH^●^ and CH_2_S, respectively.^23^ Analogous CID fragmentation products were found for [GlyCysArg]^● + 38^. Observed CID products of the radical cation of the Cys amino acid were HOCO^●^, CH_2_S, CH_2_SH^●^, and H_2_S,^35^ while Cys‐containing peptide radicals expel SH^●^ and CH_2_S.^35^ Theoretical investigations suggested that loss of a HOCO^●^ radical is energetically favored for the amino acid, followed by loss of the thiol radical and loss of the side chain (CH_2_SH^●^).^32^, ^33^, ^34^

**Scheme 2:**
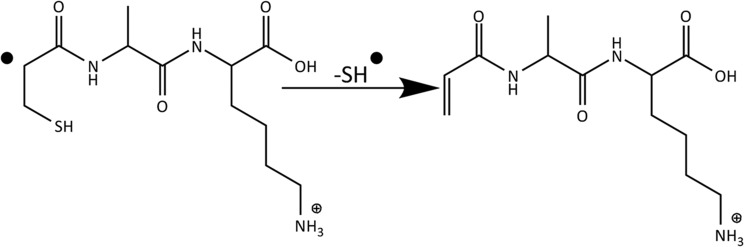
*z*‐Type electron transfer dissociation fragments expected for *z*‐ions with a Cys residue at the cleavage site are not observed. Instead, a *w*‐type ion lower in mass by 33 Da is observed due to additional neutral loss of a thiol radical. These *w*‐type ions have generally been hypothesized to possess a vinyl group at the fragment's N‐terminus

Although the vinyl structure of the *w*‐type fragment ion has often been assumed (see Scheme 2), experimental verification of this structure is scarce. Here, we use infrared multiple‐photon dissociation (IRMPD) spectroscopy^41^, ^42^, ^43^, ^44^, ^45^, ^46^, ^47^, ^48^ to experimentally confirm their structure for two *z*‐33 ETD fragments from Cys containing peptides. Several previous reports have made use of IR and UV photodissociation action spectroscopy methods to characterize ETD and ECD product ion structures.^41^, ^47^, ^48^, ^49^, ^50^, ^51^, ^52^, ^53^, ^54^, ^55^, ^56^, ^57^

## 2 EXPERIMENTAL AND COMPUTATIONAL METHODS

### 2.1 IRMPD spectroscopy

The peptides AAAACAK and AAACAAK were purchased from BIOMATIK, Canada, and used without further purification. Doubly protonated peptide ions were generated by electrospray ionization from 10^−7^‐M solutions in 50:50 acetonitrile : water with 0.5% formic acid and mass selectively stored in a modified 3‐D quadrupole ion trap mass spectrometer (Bruker, AmaZon Speed ETD).^42^, ^48^ The *z* ^*•*^‐ and *w*‐type fragment ions were produced via an ion‐ion reaction with the fluoranthene radical anion for 300 ms. Ions of interest were then mass isolated in the trap, and their IRMPD spectra were measured using the free electron laser FELIX.^58^, ^59^ Ions were irradiated by two macropulses, each pulse having an energy of 10 to 60 mJ, at a repetition rate of 10 Hz and a bandwidth of ~0.5% of the center frequency. Relating parent and fragment ion intensities as the fragmentation yield (ΣI [fragment ions]/ΣI [parent + fragment ions]), and plotting the yield as a function of laser frequency generates an infrared spectrum. The yield is linearly corrected for frequency‐dependent variations in the laser power, and the infrared frequency is calibrated using a grating spectrometer.

### 2.2 Computational chemistry

Optimized molecular geometries and theoretical infrared spectra were obtained using density functional theory (DFT) calculations as implemented in Gaussian 09 revision D01.^60^ The potential energy surface was explored to locate the lowest energy minima by using a molecular mechanics/molecular dynamics approach employing AMBER 12.^61^ Within AMBER an initial geometry optimization has been performed, followed by a simulated annealing procedure up to 500 K yielding 500 structures. These structures were reduced to about 20 to 30 candidate structures by considering their structural similarity setting appropriate rms criteria. In the final step, these candidate structures were optimized using DFT and their harmonic vibrational spectra were calculated. All computed harmonic vibrational frequencies were scaled by 0.975 and convoluted with a 25 cm^−1^ full width at half maximum Gaussian line shape to facilitate comparison with experimental spectra. Structure optimization, thermodynamic corrections, and frequency calculations were performed using B3LYP/6‐31++G(d,p). Geometries and relative energies of conformers were verified at the M06‐2X/6‐31++G(d,p) level of theory. Single‐point electronic energies were also obtained at the MP2(full)/6‐31 + G(d,p)//B3LYP/6‐31++G(d,p) level for comparison. The computational procedure is described in more detail elsewhere.^43^, ^62^, ^63^

## 3 RESULTS AND DISCUSSION

**Figure 1:**
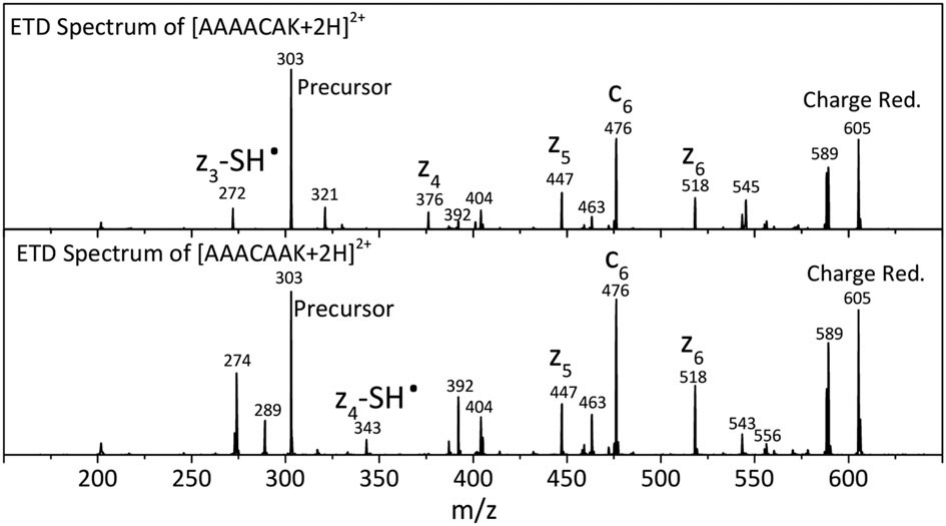
Electron transfer dissociation (ETD) mass spectra of (top) [AAAACAK + 2H]^2+^ and (bottom) [AAACAAK + 2H]^2+^. ETD fragment ions are annotated. Neutral loss of an SH^•^ radical is observed for the z_3_^•^ fragment from AAAACAK and for the z_4_^•^ ion from AAACAAK, ie, from *z* ^•^‐ions with the Cys residue at the cleavage site

Figure 1 shows the ETD mass spectra for the two doubly protonated heptapeptides AAAACAK and AAACAAK investigated in this study, having a Cys residue in the third and fourth position from the C‐terminus, respectively. The identified ETD fragment ions are annotated in Figure 1. As a consequence of the basic Lys residue at the C‐terminus, the series of *z* ^•^‐type fragment ions are nearly complete, except for the smallest ions.

Interestingly, for AAAACAK—with Cys in the third position from the C‐terminus—the z_3_ fragment is missing, but instead, a peak 33 Da lower in mass is observed. Analogously, for AAACAAK, the *z*_4_ ion is missing and instead a peak at the nominal mass of *z*_4_–33 is observed. We observe that neutral loss of a thiol radical (SH^•^) occurs only for *z* ^*•*^‐ions that contain a cysteine residue at the cleavage site; these fragments are commonly referred to as *w*‐type ions. For the longer *z* ^*•*^ *‐*ions, which do contain a Cys residue but not at the cleavage site; SH^•^ loss is not observed upon ETD. However, isolating one of these *z* ^•^‐ions and applying CID in an MS^3^ experiment did result in expulsion of neutral fragments of 33 and 46 Da, the latter likely corresponding to loss of CH_2_S (see Figures S1 and S2).

The *z* ^•^‐type ETD fragment ions that underwent neutral loss of 33 Da were investigated using IRMPD spectroscopy to determine their molecular structures. Upon IRMPD of the z_3_‐33 ETD fragment ion, ie, the *w*_3_‐ion, dissociation is observed into *m*/*z* 147 (*y*_1_‐ion), *m*/*z* 129 (Lys‐residue), and *m*/*z* 84 (fragment of Lys residue). For the *w*_4_ ETD fragment ion, IRMPD leads to fragmentation channels *m*/*z* 218 (*y*_2_‐ion), *m*/*z* 147 (*y*_1_‐ion), and *m*/*z* 129 (Lys‐residue). IRMPD spectra are constructed including fragmentation into all of these mass channels.

**Figure 2:**
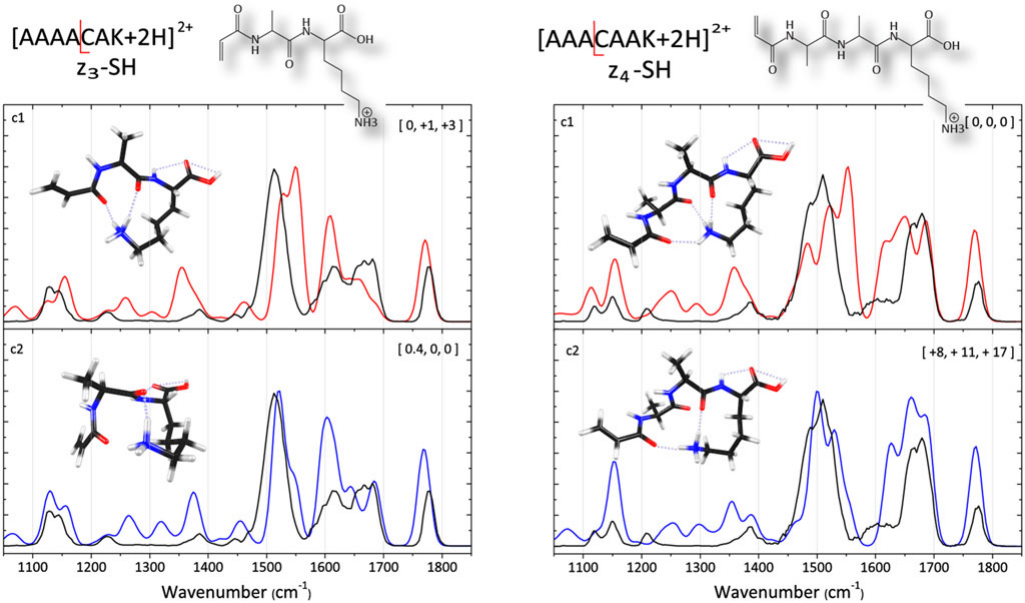
Infrared multiple‐photon dissociation spectra of the (left column) z_3_‐33 electron transfer dissociation (ETD) fragment (*w*_3_) from [AAAACAK+2H]^2+^ at *m*/*z* 272 (black) and of the (right column) z_4_‐33 ETD fragment (*w*_4_) of [AAACAAK+2H]^2+^ at *m*/*z* 343 (black). The experimental spectra are compared with predicted spectra for the lowest energy structures (red) and to the qualitatively best matching calculated spectra (blue). Relative energies (kJ/mol) at the B3LYP, M06‐2X, and MP2(full) levels of theory are given in the square brackets

The left panel of Figure 2 shows the IRMPD spectrum of the *w*_3_ fragment ion from [AAAACAK+2H]^2+^ at *m*/*z* 272, and that of the *w*_4_ fragment ion from [AAACAAK+2H]^2+^ at *m*/*z* 343 is shown in the right panel. For both these z_3_‐33 and the z_4_‐33 ions, computed spectra for a structure containing a vinyl group at the N‐terminus match favorably with the experimental spectra. In this structure, the radical thiol group is expelled from the cysteine side chain and a double bond is formed between the C_α_ and C_β_ atoms at the site of cleavage. The calculated spectra of the lowest energy conformers (red) and the best matching conformers (blue), from a visual inspection, are presented. Computed spectra for additional conformers of these species are shown in the Figure S3.

For the w_3_‐ion of [AAAACAK+2H]^2+^, the calculated spectra of two effectively iso‐energetic conformers c1 and c2 are shown in the two panels on the left in Figure 2. Both conformers are compact structures where the protonated Lys amine group hydrogen‐bonds with the two amide carbonyl oxygen atoms of the backbone. The band near 1770 cm^−1^ is attributed to the carbonyl stretch of the C‐terminal carboxylic acid group. In the computed spectra of both conformers, the unresolved feature between 1600 and 1700 cm^−1^ consists of four relatively strong bands having amide C═O stretch character mixed with ammonium bending character. In addition, the band near 1660 cm^−1^ in both conformers has significant vinyl C═C stretch character mixed in. The intense feature just above 1500 cm^−1^ is due to three vibrational modes, symmetric ammonium bending and the two amide NH bending modes. The c1 calculation attributes the highest intensity to the ammonium bending mode near 1550 cm^−1^, while the c2 calculation attributes the highest intensity to the amide NH bending modes around 1520 cm^−1^, leading to a better match for c2. The band at 1355 cm^−1^ in c1 (mainly delocalized CH bending character) is shifted to 1375 cm^−1^ in conformer c2, the latter being in slightly better agreement with experiment.

To the right in Figure 2, two different conformers for the vinyl group containing structure of the *w*_4_‐ion from [AAACAAK+2H]^2^ are shown along with their predicted IR spectra. The lowest‐energy conformer (c1) in the top panel has its protonated Lys amine group hydrogen‐bonded with all three amide oxygen atoms. The bottom panel displays a higher‐energy conformer (c2) in which the ammonium group is hydrogen‐bonded with just two amide oxygen atoms; the central amide moiety in the molecule is not engaged in hydrogen bonding. This leads to differences in the two predicted spectra around 1650 to 1600 cm^−1^ and 1550 to 1480 cm^−1^. Although higher in energy at all levels of theory considered here, the c2 conformer qualitatively provides a better spectral match, and we suggest to assign this structure.

The band near 1770 cm^−1^ is again the C‐terminal COOH carbonyl stretch. The feature roughly observed between 1600 and 1700 cm^−1^, including the shoulder on the low‐frequency side, is due to several normal modes mixing amide CO stretch character with asymmetric bending of the Lys NH_3_^+^ group. Again, the vinyl C═C stretch is mixed with the CO stretch of the adjacent amide and this mode appears at 1622 cm^−1^ in c2 and 1635 cm^−1^ in c1, at the position of the shoulder in the experimental spectrum. The c1 calculation predicts a strong, quite red‐shifted CO stretch/NH_3_^+^ bending mode at 1613 cm^−1^, which causes the c1 conformer to match poorly with experiment. Also the amide II experimental feature around 1500 cm^−1^ matches better with the computed spectrum of c2 than with that of c1. In both spectra, the feature comprises four intense bands, the symmetric NH_3_^+^ bending mode and three amide NH bending modes. The ammonium bending mode is predicted near 1550 cm^−1^ for both conformers, but is 4 times more intense in c1 than in c2, explaining the better match with experiment for c2. The remainder of the spectrum is analogous to that of the w_3_‐ion, with the C‐terminal COH bending mode near 1150 cm^−1^ as the most prominent peak.

Secondary neutral loss from *z* ^*•*^‐type ions from ExD—and thiol loss from *z* ^*•*^‐type ions containing Cys residues in particular—leading to *w*‐type ions has been addressed in various investigations.^14^, ^16^, ^18^, ^21^, ^31^ The structure of *w*‐type ions is commonly assumed to involve a double bond between the α‐ and β‐carbon atoms of the residue at the cleavage site. This fragment ion structure was hypothesized on the basis of differences in the CID MS/MS behavior of *z* ^*•*^‐ions versus (*z* + 1)‐ions.^20^ Related, the differences in bond dissociation energies for H‐atom removal from the α and β carbons have been used to rationalize the CC double bond structure of the *w*‐ion.^16^, ^21^ Computational investigations of the potential energy surface of the dissociation reaction at the DFT level also indicate the formation of a vinyl group at the N‐terminus of the *w*‐ion.^27^ However, direct experimental probing of the *w*‐ion structure has until now not been reported to our knowledge. In this contribution, IR spectroscopy of two *w*‐type ions formed by loss of an SH‐radical from a Cys residue at the cleavage site confirms the vinyl group structure suggested previously.^14^, ^16^, ^18^, ^21^, ^27^, ^31^ Indirectly, the observation of this structure also confirms the suggested charge remote fragmentation pathways suggested.^21^, ^22^, ^23^, ^27^, ^31^

Other *z* ^*•*^‐type ions were observed to expel a thiol radical only upon applying additional CID activation after ETD, in line with reported higher rates of formation of *w*‐ions compared with *u*‐ions.^18^ Also in accordance with previous studies,^27^, ^29^ loss of CH_2_S (46 Da) after collisional activation was only observed for *z* ^*•*^‐ions that contain a Cys residue, but not at the cleavage site. Spectroscopic probing of the structure of these fragment ions is challenging because of the more involved ETD/CID MS^n^ scheme required to produce them, and the inherent lower ion yields as compared with the *w*‐type ions; such experiments are reserved for future studies.

## 4 CONCLUSION

We have reported the first infrared spectra for *w*‐type fragment ions resulting from ETD of two Cys‐containing peptides. Specifically, the mass peak at *m*/*z* 272 in the ETD mass spectrum of [AAAACAK+2H]^2+^ and that at *m*/*z* 343 in the ETD mass spectrum of [AAACAAK+2H]^2+^, which cannot be assigned to sequence ions but are the result of neutral SH^●^‐loss from the radical z_3_ and z_4_‐ions of the respective peptides, have been investigated with infrared ion spectroscopy. Evaluation of experimental and predicted IR spectra confirm that these *w‐*type fragment ions possess a structure having a C═C double bond at their N‐terminus. This structure supports the mechanism proposed for the formation of *w*‐type fragment ions in ExD, proceeding through cleavage of the C_β_‐S bond of the Cys residue with concomitant formation of a double bond between the C_α_ and C_β_ atoms of the *w*‐ion. Elimination of the SH^●^‐group is only observed for *z* ^*•*^‐ions with a cysteine residue at the cleavage site, independent of its position of cysteine in the peptide.

## Supporting information

Figure S1. Mass spectra of CID applied on *z*•‐ions obtained from ETD of [AAAACAK+2H]2+. The mass peaks corresponding to loss of the radical thiol group are indicated.
Figure S2. Mass spectra upon CID fragmentation of *z*•‐ions obtained by ETD on [AAACAAK+2H]2+. The mass peaks corresponding to loss of the radical thiol group are indicated.
Figure S3. Additional conformers and their computed IR spectra of the z3‐33 and z4‐33 ions under discussion.
