# Supplementary material for: w‐Type ions formed by electron transfer dissociation of Cys‐containing peptides investigated by infrared ion spectroscopy
Source: J Mass Spectrom. 2018 Nov 12;53(12):1207–13. doi: 10.1002/jms.4298 (PMC6283004; doi:10.1002/jms.4298)
Supplement: Supplementary file 1 — Figure S1. Mass spectra of CID applied on z•‐ions obtained from ETD of [AAAACAK+2H]2+. The mass peaks corresponding to loss of the radical thiol group are indicated. Figure S2. Mass spectra upon CID fragmentation of z•‐ions obtained by ETD on [AAACAAK+2H]2+. The mass peaks corresponding to loss of the radical thiol group are indicated. Figure S3. Additional conformers and their computed IR spectra of the z3‐33 and z4‐33 ions under discussion. [file JMS-53-1207-s001.pdf]

w-type ions formed by Electron Transfer Dissociation of Cys-containing peptides investigated by infrared ion spectroscopy

Lisanne J.M. Kempkes[1], Jonathan Martens[1], Giel Berden[1], Jos Oomens[1,2,*]

[1] *Radboud University, Institute for Molecules and Materials, FELIX Laboratory, Toernooiveld 7c, 6525 ED Nijmegen, The Netherlands*

[2] *Van't Hoff Institute for Molecular Sciences, University of Amsterdam, Science Park 904, 1098 XH Amsterdam, The Netherlands*

* Corresponding author: j.oomens@science.ru.nl

Supporting Information

Additional figures: CID mass spectra of $z^{\bullet}$-type ETD fragment ions containing a Cys residue which is not at the cleavage site. The $z^{\bullet}$-type fragments with Cys at the cleavage site are discussed in the main text. Computed spectra for additional conformers of the vinyl-group N-terminal structures of the z_3 – 33 and z_4 – 33 ions presented in Figure 2 of the main text.

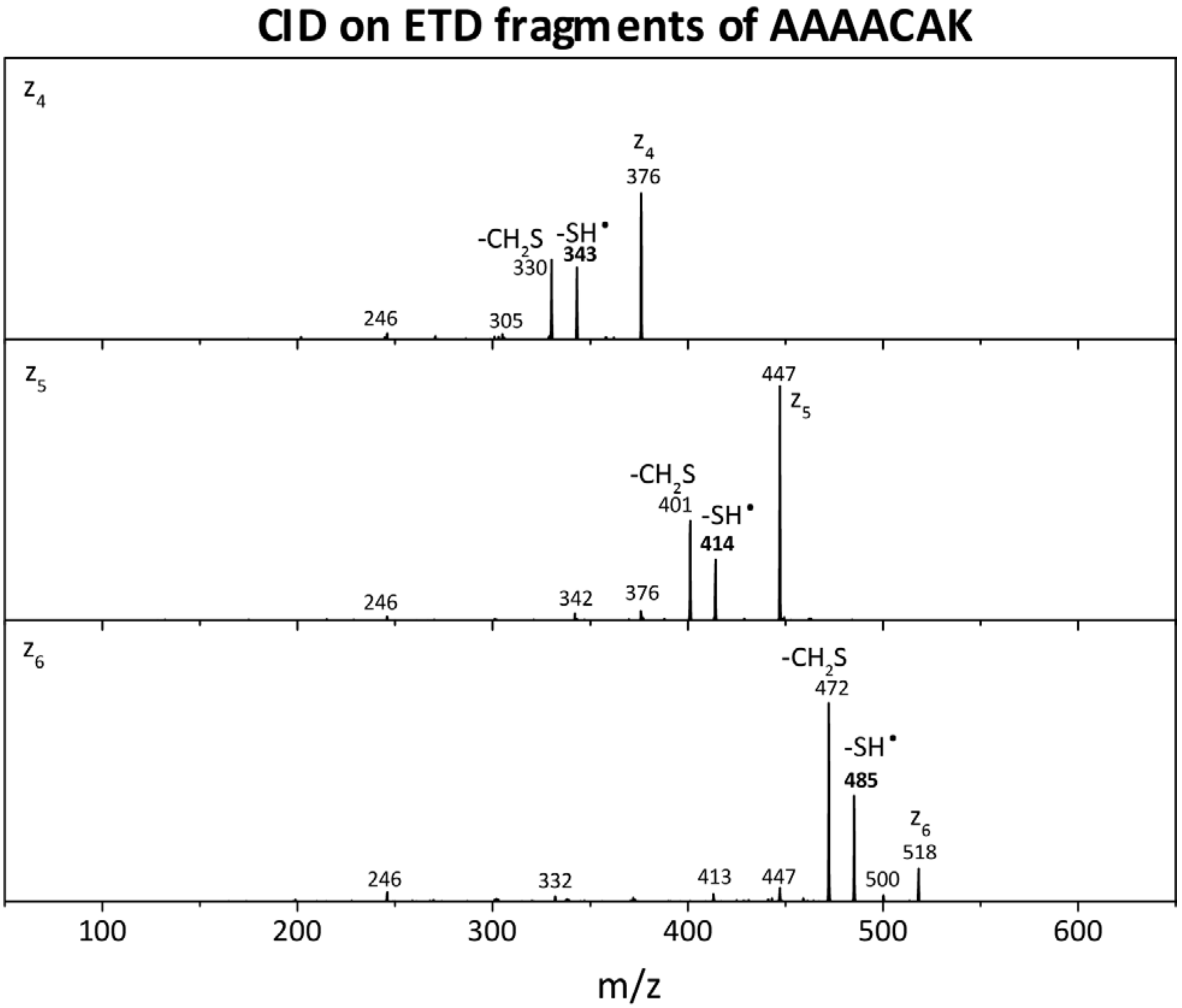

Figure S1. *Mass spectra of CID applied on z•-ions obtained from ETD of [AAAACAK+2H]$^{2+}$. The mass peaks corresponding to loss of the radical thiol group are indicated.*

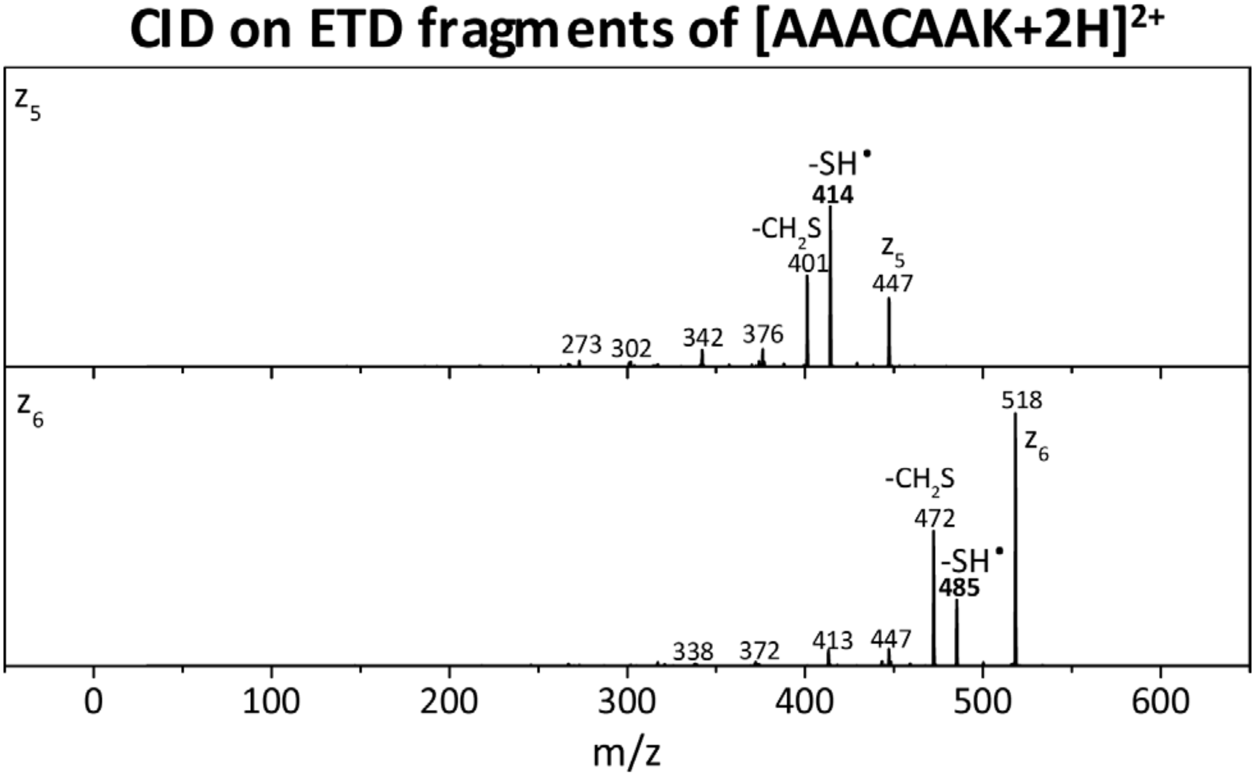

Figure S2. *Mass spectra upon CID fragmentation of z•-ions obtained by ETD on [AAACAAK+2H]$^{2+}$. The mass peaks corresponding to loss of the radical thiol group are indicated.*

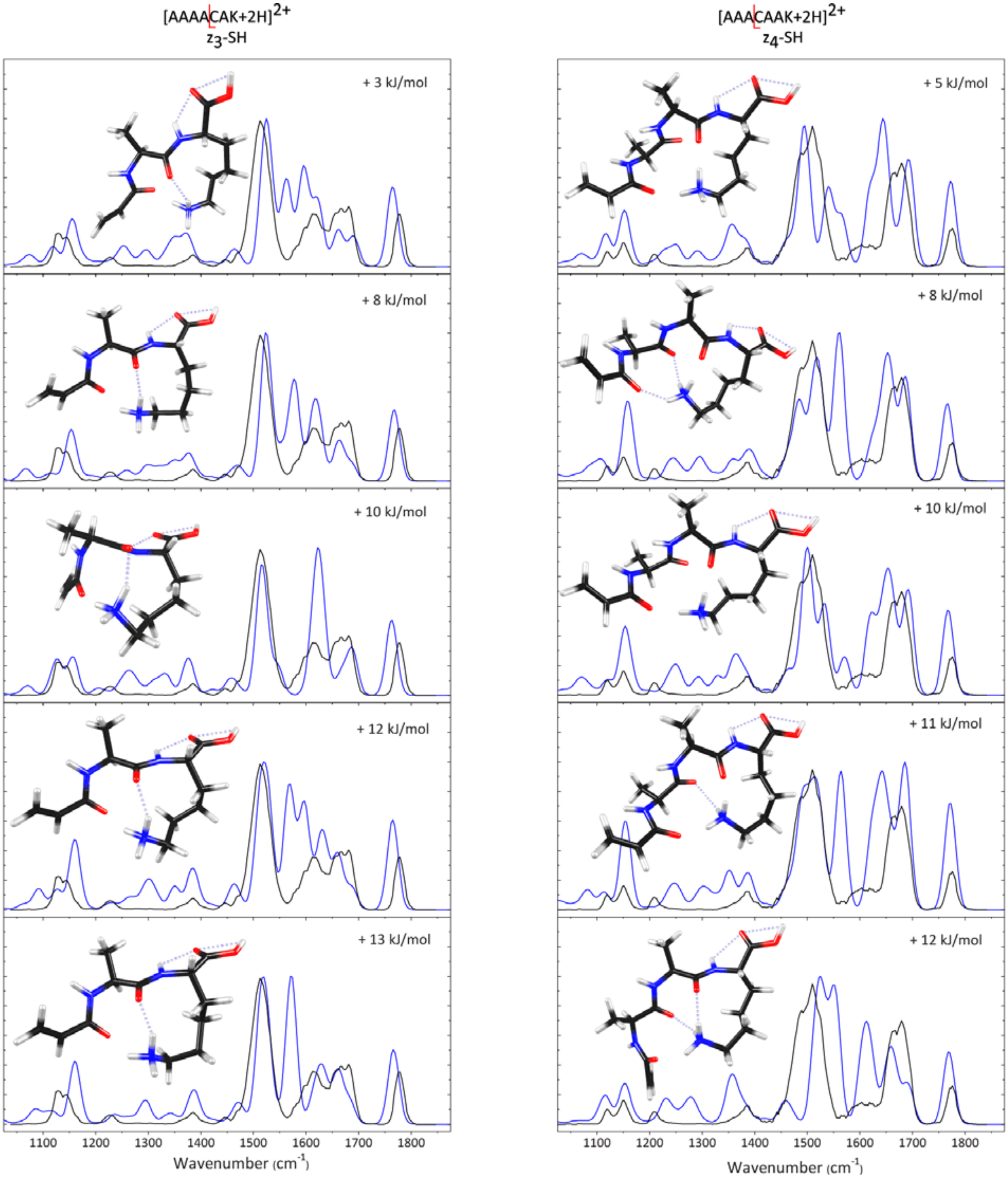

Figure S3. *Additional conformers and their computed IR spectra of the $z_3 - 33$ and $z_4 - 33$ ions under discussion.*
